# Presentation of Sex Chromosomal Disorders of Sex Development With Genital Ambiguity: A Case Report on a Rare Medical Condition

**DOI:** 10.7759/cureus.67496

**Published:** 2024-08-22

**Authors:** Srinija Garlapati, Shailaja V Mane, Supriya Gupte, Sajili Mehta, Aryan Gupta, Om Prasanth Reddy Avuthu

**Affiliations:** 1 Department of Pediatrics, Dr. D. Y. Patil Medical College, Hospital and Research Centre, Dr. D. Y. Patil Vidyapeeth (Deemed to be University), Pune, IND; 2 Department of Pediatric Endocrinology, Dr. D. Y. Patil Medical College, Hospital and Research Centre, Dr. D. Y. Patil Vidyapeeth (Deemed to be University), Pune, IND; 3 Department of Pediatric Neurology, Dr. D. Y. Patil Medical College, Hospital and Research Centre, Dr. D. Y. Patil Vidyapeeth (Deemed to be University), Pune, IND

**Keywords:** klinefelter syndrome, hypospadiasis, bifid scrotum, hcg stimulation test, karyotyping, ambiguous genitalia

## Abstract

Klinefelter syndrome (KS; XXY syndrome) is a common chromosomal abnormality associated with various physical and developmental characteristics. It rarely presents with ambiguous genitalia, a feature more typical of disorders of sex development (DSDs). Here, we describe a case of a five-month-old male infant with 47,XXY karyotype who presented with ambiguous genitalia which include bifid scrotum, small phallus, and penoscrotal hypospadias. Initial anthropometry and ultrasound evaluations were followed by hormonal and genetic analyses. Elevated follicle-stimulating hormone and low testosterone levels led to further testing, including a human chorionic gonadotropin stimulation test and karyotyping, which confirmed 47,XXY KS. This case underscores the need for thorough genetic evaluation in infants presenting with ambiguous genitalia, highlighting that KS can present with features overlapping DSDs. Comprehensive diagnostic approaches combining genetic, endocrinological, and clinical assessments are crucial for accurate diagnosis and management. This case aims to raise awareness among paediatricians about the potential for atypical genital presentations in KS and the importance of karyotype analysis in such scenarios.

## Introduction

Klinefelter syndrome (KS; XXY syndrome) is a relatively common chromosomal abnormality, affecting approximately one in 500 to 1,000 live births. It is characterized by tall stature, learning difficulties, gynecomastia, and an altered ratio between the upper and lower body segments. Affected individuals often have hypotonia, clinodactyly, and hypertelorism. Testicular abnormalities include small testes with low-normal androgen production, leading to defective spermatogenesis and Sertoli cell function, resulting in infertility. Other genital abnormalities include a small phallus and a higher incidence of hypospadias and cryptorchidism. KS is relatively common, occurring in approximately one in 600 males with the 47,XXY karyotype. Despite being prevalent, KS is frequently misdiagnosed, leaving many people without a diagnosis until later in life [1]. Disorders of sex development (DSDs) involve atypical development of chromosomal, gonadal, or anatomical sex. External genitalia alone are rarely conclusive for a specific diagnosis, and about 50% of 46,XY DSD cases lack a definitive diagnosis. KS can rarely present with clinical manifestations similar to DSDs.

## Case presentation

**Figure 1:**
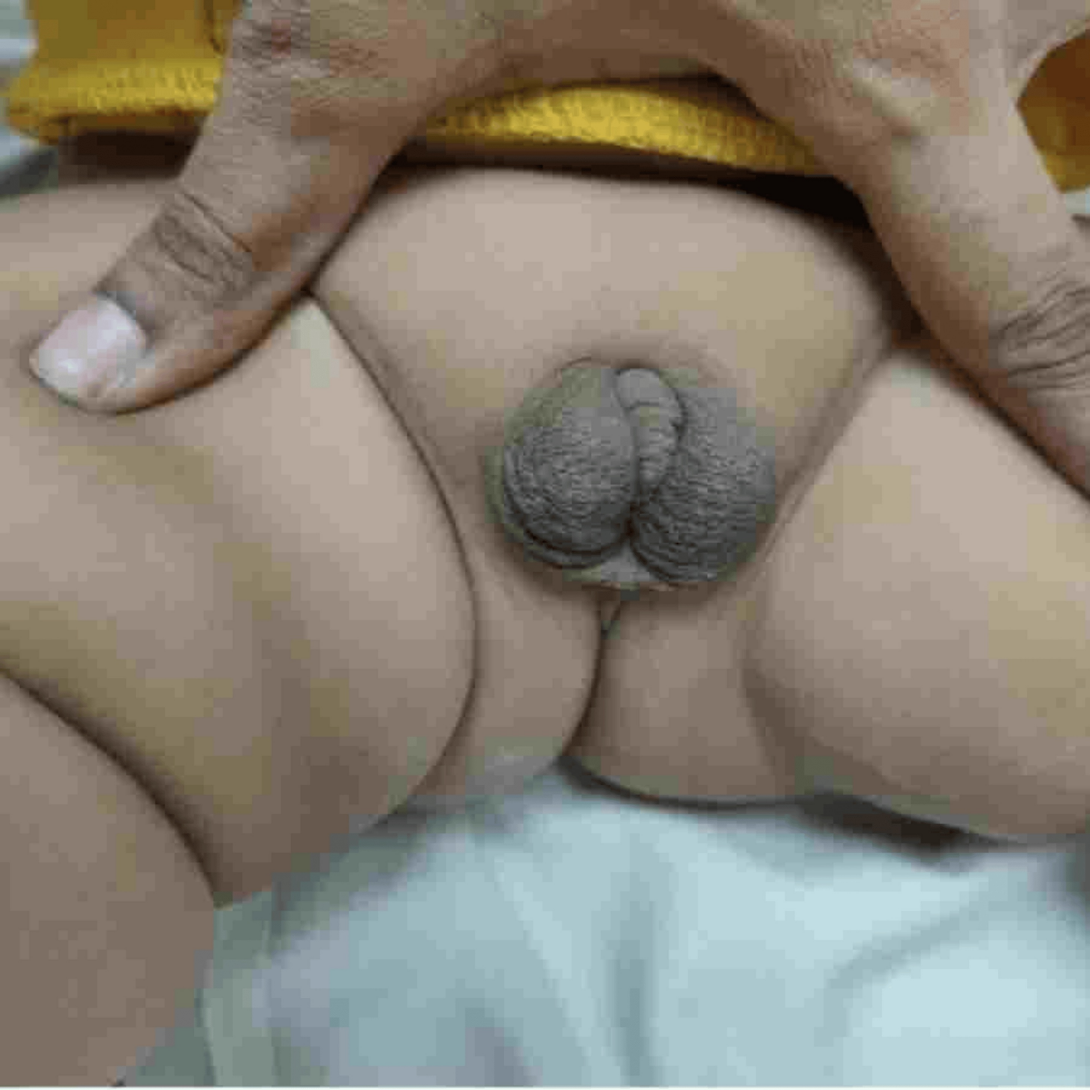
Small penis with descended testis in bifid labioscrotal folds on presentation

A five-month-old male child born into a nonconsanguineous marriage was referred to our outpatient department with the concern of abnormal genitalia. He was the fourth child in the family and has no significant family, perinatal, or developmental history. Anthropometry revealed a height of 65 cm (between the 10th and 25th centiles), a weight of 6.2 kg (between the 3rd and 10th centiles), and a head circumference of 43 cm (at the 25th centile). Physical examination of external genitalia revealed the bifid scrotum, small phallus (Figure 1) with a stretched penile length of 2.5 cm, and penoscrotal hypospadias. Ultrasound pelvis did not show any Mullerian structures, and renal ultrasound was normal. 

Laboratory analyses were conducted upon presentation (Table 1), involving screening for Congenital adrenal hyperplasia through serum cortisol level examinations. Assessment of gonadal response to gonadotropin stimulation was performed to evaluate the presence and functionality of testicular gonadal tissue.

**Table 1:** Hormonal analysis done on presentation IU: international units

Parameter	Result	Normal range
Follicle-stimulating hormone	3.87 mIU/mL	0.10-2.40 mIU/mL
Luteinizing hormone	2.3 mIU/mL	0.02-5.0 mIU/mL
Total testosterone level	40.05 ng/dL	75-400 ng/dL
Serum cortisol	13.8 mcg/dL	3.7-19.40 mcg/dL

**Figure 2:**
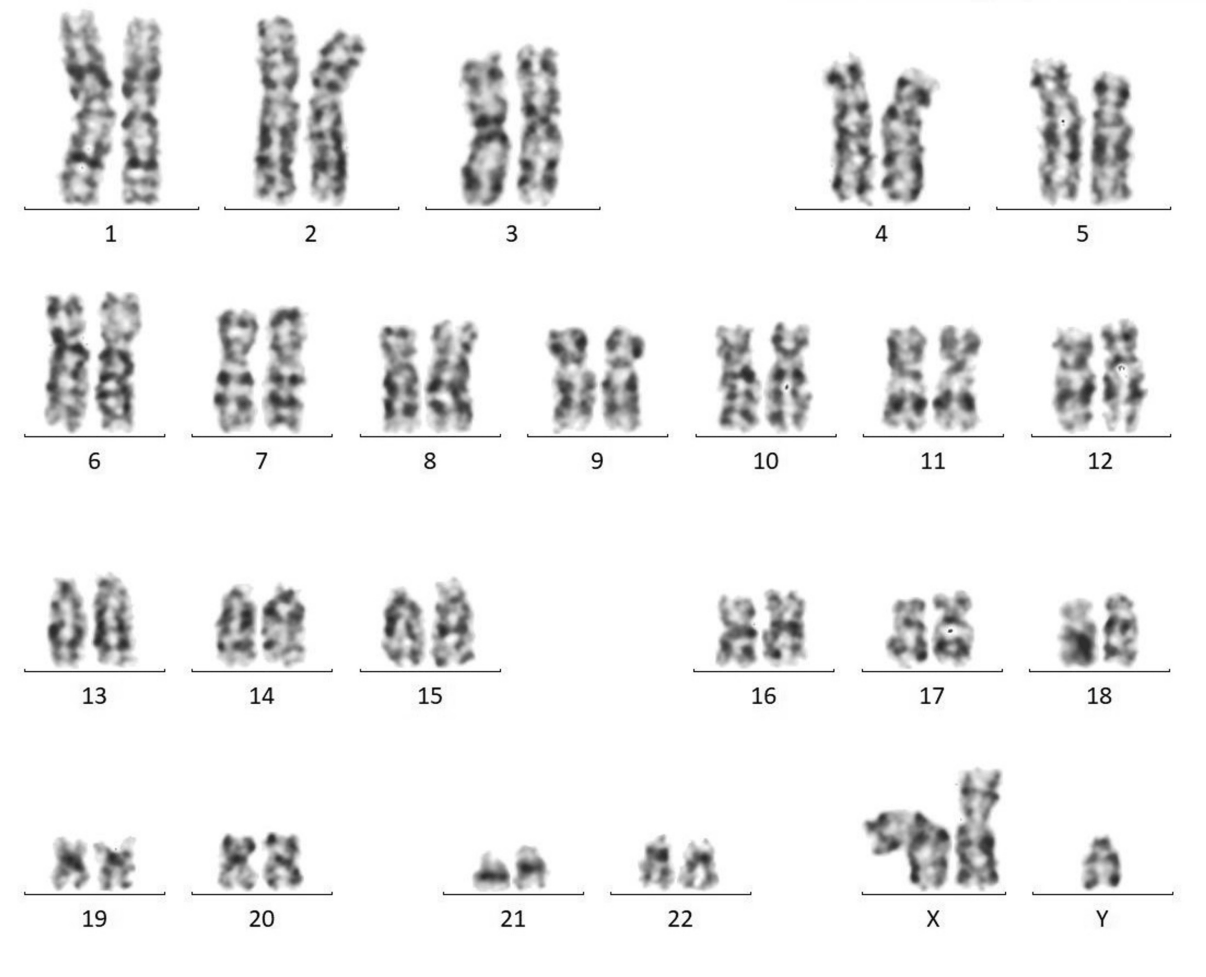
Karyotyping of the patient showing 47,XXY (resolution: 450 bphs)

The patient was subjected to a human chorionic gonadotropin (hCG) stimulation test, where 1,500 IU of hCG was given subcutaneously [2], and 72 hours later, serum samples for testosterone (T), dihydrotestosterone (DHT), and androstenedione levels were taken and assessed. The T/DHT ratio was 11, following the hCG stimulation test. A T/DHT ratio greater than 17 shows 5-alpha reductase deficiency. Therefore, 5-alpha reductase deficiency was ruled out. Karyotyping done from the source being peripheral (venous) blood showed 47,XXY KS (Figure 2).

The ratio of testosterone to androstenedione was 5:1. A ratio of testosterone to androstenedione lower than 0.8 indicates 17β-hydroxysteroid dehydrogenase III deficiency. Therefore, 17β-hydroxysteroid dehydrogenase III deficiency was ruled out.

## Discussion

DSDs represent a range of congenital conditions marked by atypical development of genital structures. These disorders can be associated with gene variations, developmental programming, and hormone levels [3]. The category of 46,XY DSD includes conditions such as abnormal testicular differentiation, defects in testosterone production, and impaired testosterone action. Sex chromosomal DSDs include Turner syndrome, KS, and 45,X/46,XY gonadal dysgenesis. Typically, Turner syndrome and KS do not present with genital ambiguity [3].

Sreejith et al. emphasize that genetic defects may not always be apparent through physical examination alone. Thus, genetic analysis is crucial for men with infertility [4]. Guerrero-Gonzalez and Estrada have done a study on 55 patients with DSDs, noting that the neonatal period was when most DSD cases were first identified (69.09%). Among these, 58.18% were male. Common genital anomalies observed included hypospadias (45.45%), cryptorchidism (21.82%), and micropenis (12.73%). Of 28 patients with karyotyping, 12 had sex chromosome DSDs, including two patients with 46,XX DSD and 14 patients with 46,XY DSD [5]. Molecular confirmation of DSDs is often limited by cost and accessibility [6].

In our case, clinical suspicion of DSDs was confirmed through karyotyping, leading to a diagnosis of KS. Khanna et al. have highlighted that while clinical history and examination are foundational for diagnosing DSDs, they should be complemented by modern diagnostic tools such as karyotyping, imaging, hormonal assays, and gonadal biopsy or endoscopy/laparoscopy [7].

## Conclusions

This case highlights the complexity of diagnosing and managing ambiguous genitalia in infants. A comprehensive approach that integrates genetic, endocrinological, surgical, and psychological perspectives is essential for optimal care and support of the affected individual and their family. This is to increase awareness among pediatricians about the atypical presentation of genitals in the case of KS, and this case report highlights the importance of karyotype assessment in all atypical genitalia. The patient has received medical treatment, undergone genetic counseling, and has been referred to pediatric surgery. He is currently under regular follow-up care.
